# A Rare Myelin Protein Zero (*MPZ*) Variant Alters Enhancer Activity *In Vitro* and *In Vivo*

**DOI:** 10.1371/journal.pone.0014346

**Published:** 2010-12-16

**Authors:** Anthony Antonellis, Megan Y. Dennis, Grzegorz Burzynski, Jimmy Huynh, Valerie Maduro, Chani J. Hodonsky, Mehrdad Khajavi, Kinga Szigeti, Sandeep Mukkamala, Seneca L. Bessling, NISC Comparative Sequencing Program, William J. Pavan, Andrew S. McCallion, James R. Lupski, Eric D. Green

**Affiliations:** 1 Department of Human Genetics, University of Michigan Medical School, Ann Arbor, Michigan, United States of America; 2 Department of Neurology, University of Michigan Medical School, Ann Arbor, Michigan, United States of America; 3 Genome Technology Branch, National Human Genome Research Institute, National Institutes of Health, Bethesda, Maryland, United States of America; 4 McKusick–Nathans Institute of Genetic Medicine and Department of Molecular and Comparative Pathobiology, The Johns Hopkins University School of Medicine, Baltimore, Maryland, United States of America; 5 Department of Molecular and Human Genetics, Houston, Texas, United States of America; 6 Department of Neurology, Houston, Texas, United States of America; 7 NIH Intramural Sequencing Center (NISC), National Human Genome Research Institute, National Institutes of Health, Bethesda, Maryland, United States of America; 8 Genetic Disease Research Branch, National Human Genome Research Institute, National Institutes of Health, Bethesda, Maryland, United States of America; 9 Department of Pediatrics, Baylor College of Medicine, Houston, Texas, United States of America; 10 Texas Children's Hospital, Houston, Texas, United States of America; Deutsches Krebsforschungszentrum, Germany

## Abstract

**Background:**

Myelin protein zero (MPZ) is a critical structural component of myelin in the peripheral nervous system. The *MPZ* gene is regulated, in part, by the transcription factors SOX10 and EGR2. Mutations in *MPZ*, *SOX10*, and *EGR2* have been implicated in demyelinating peripheral neuropathies, suggesting that components of this transcriptional network are candidates for harboring disease-causing mutations (or otherwise functional variants) that affect *MPZ* expression.

**Methodology:**

We utilized a combination of multi-species sequence comparisons, transcription factor-binding site predictions, targeted human DNA re-sequencing, and *in vitro* and *in vivo* enhancer assays to study human non-coding *MPZ* variants.

**Principal Findings:**

Our efforts revealed a variant within the first intron of *MPZ* that resides within a previously described SOX10 binding site is associated with decreased enhancer activity, and alters binding of nuclear proteins. Additionally, the genomic segment harboring this variant directs tissue-relevant reporter gene expression in zebrafish.

**Conclusions:**

This is the first reported *MPZ* variant within a cis-acting transcriptional regulatory element. While we were unable to implicate this variant in disease onset, our data suggests that similar non-coding sequences should be screened for mutations in patients with neurological disease. Furthermore, our multi-faceted approach for examining the functional significance of non-coding variants can be readily generalized to study other loci important for myelin structure and function.

## Introduction

Myelin protein zero (MPZ) is an integral membrane glycoprotein and a major structural component of peripheral nervous system myelin. MPZ has cell-adhesion properties and forms tetramers that are thought to bind other tetramers on opposing membranes, thus holding concentric layers of myelin intact [1], [2], [3], [4]. The importance of MPZ for proper myelination is underscored by the fact that *MPZ* protein-coding and splice-site mutations cause demyelinating Charcot-Marie-Tooth disease type 1B (CMT1B) [5], [6], [7]; however, mutations in transcriptional regulatory elements have not been reported to date. The identification of null *MPZ* alleles in patients with CMT1B suggests that there is a threshold in the amount of MPZ required for proper myelination [8]; in support of this notion, mice that are heterozygous for an *Mpz*-knockout allele develop a late-onset peripheral demyelinating neuropathy [9]. It is thus reasonable to consider cis-acting regulatory elements as excellent candidates for harboring mutations that affect *MPZ* transcription.

The regulation of *MPZ* transcription involves at least two previously described regulatory elements: a proximal promoter and an enhancer located in the first intron [10], [11], [12]. Two transcription factors with known roles in *MPZ* expression are SRY-box 10 (SOX10) and early growth response 2 (EGR2); indeed, mutations in each of these genes have been shown to cause CMT disease [8], [13]. Other studies have demonstrated that SOX10 and EGR2 bind to the intronic enhancer both *in vitro* and *in vivo*, and that mutagenesis of the SOX10-binding sites within this enhancer reduces binding of these transcription factors [11], [14], [15], [16], [17]. Such findings suggest that variants within these SOX10- and EGR2-binding sites may affect *MPZ* transcriptional regulation. In support of this notion, a CMT-associated mutation has been identified in a SOX10 binding site at the gap junction protein beta 1 (*GJB1*) locus [18], [19].

We sought to identify and study *MPZ* variants within evolutionarily conserved SOX10- and EGR2-binding sites. In this study, we report a rare human variant that alters the activity of an *MPZ* enhancer, a finding that has implications for understanding the transcriptional regulation of *MPZ* and for assessing the functional significance of non-coding variants within genes implicated in human inherited neuropathies.

## Results

### Conserved sequences and transcription factor-binding sites within *MPZ*

**Figure 1:**
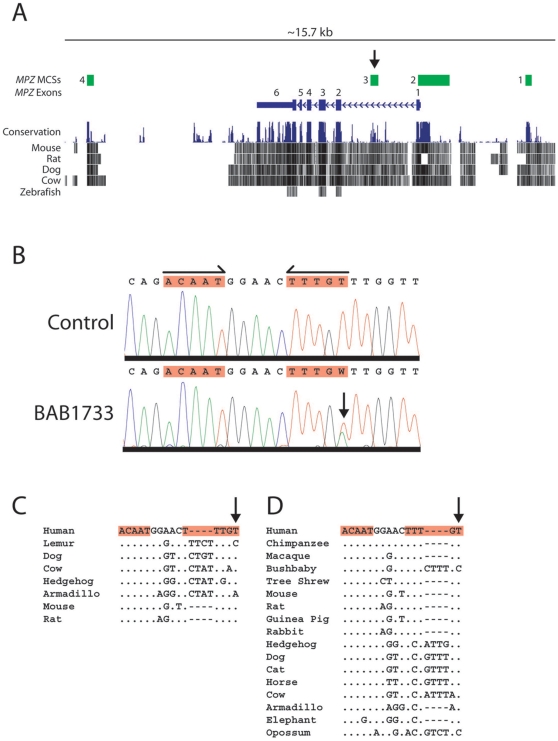
Analysis of MCSs in the genomic region encompassing *MPZ*. (**A**) Representation of the *MPZ* gene and its flanking regions from the UCSC Human Genome Browser. From top to bottom, tracks are shown displaying information about *MPZ*-MCSs, *MPZ* exons, overall multi-species sequence conservation, and pair-wise sequence conservation between human and five other vertebrate species. *MPZ*-MCS3 within *MPZ* intron 1 is indicated by an arrow. (**B**) Sequence chromatograms for a region within *MPZ*-MCS3 are shown for an unaffected individual (control) and patient BAB1733. Two monomeric consensus SOX10-binding sequences in a head-to-head configuration are indicated by red highlights and horizontal arrows. The position of the c.126-1086T>A variant (heterozygous in the patient) is indicated with a vertical arrow. (**C, D**) Multi-sequence alignments of the consensus SOX10-binding sequences within *MPZ*-MCS3 (highlighted in red) are shown containing sequences from the indicated species, as generated by MultiPipMaker (**C**) or available on the UCSC Human Genome Browser (**D**). The position of c.126-1086T in each alignment is indicated by a vertical arrow; dots indicate bases that are identical to the human reference sequence and dashes represent gaps within the alignments.

We used two approaches to identify evolutionarily conserved sequences [i.e., multi-species conserved sequences (MCSs)] within the *MPZ* gene. First, we generated and collected sequences of the genomic region encompassing *MPZ* from multiple species, generated a multi-sequence alignment, and then analyzed that alignment for highly conserved segments. Second, we examined this genomic region on the UCSC Human Genome Browser [20], which contains annotations about evolutionary sequence conservation. These analyses revealed four MCSs of particular interest: *MPZ*-MCS1, *MPZ*-MCS2, *MPZ*-MCS3, and *MPZ*-MCS4 (Fig. 1A). Because SOX10 and EGR2 play important roles in *MPZ* expression, we computationally searched for predicted SOX10- and EGR2-binding sites that reside within each *MPZ*-MCS (see Methods for details). This analysis revealed a total of 14 SOX10- and four EGR2-binding sites in the four MCSs (Table 1). For the experiments described below, each MCS tested directly corresponds to the UCSC Genome Browser coordinates provided in the second column of Table 1.

**Table 1:** Transcription factor-binding sites within *MPZ* MCSs.

*MPZ*-MCS	MCS Position^1^	SOX10-binding Site^1,2^	EGR2-binding Site^1^
*MPZ*-MCS1	chr1:159549725-159549930	chr1:159549894-159549898	
		chr1:159549816-159549820	
		chr1:159549756-159549760	
*MPZ*-MCS2	chr1:159546302-159547303	chr1:159547247-159547251	chr1:159546546-159546554
		chr1:159547231-159547235	
		chr1:159547177-159547181	
		chr1:159547044-159547048	
		chr1:159546979-159546983	
		chr1:159546648-159546652	
		**chr1:159546573-159546588**	
*MPZ*-MCS3	chr1:159544784-159545029	**chr1:159544923-159544937**	chr1:159544903-159544911
		chr1:159544891-159544895	chr1:159544853-159544861
*MPZ*-MCS4	chr1:159535703-159535928	chr1:159535756-159535760	chr1:159535795-159535803
		chr1:159535734-159535738	

1 Coordinates are from the March 2006 assembly of the human genome sequence at the UCSC Genome Browser.

2 Dimeric SOX10-binding sites are indicated in bold.

### Identification of the c.126-1086T>A nucleotide variant in *MPZ*-MCS3

As part of a larger effort to screen patients with neurological disease for genetic variants, we sequenced the above four MCSs (as well as all *MPZ* protein-coding regions) in 192 individuals, including 69 patients diagnosed with demyelinating peripheral neuropathy. These efforts revealed one novel variant (c.126-1086T>A) in a heterozygous state within *MPZ*-MCS3 (which resides in the first intron of *MPZ*; see Fig. 1A) in one patient from our cohort with congenital non-progressive central nervous system hypomyelination affecting the supratentorial structures, but no signs of demyelinating peripheral neuropathy (BAB1733; Fig. 1B). Importantly, this variant resides within a previously-described SOX10-binding site within *MPZ*-MCS3 (Fig. 1B, black half-arrows and red box) [11], [16]; dimerized SOX10 proteins are known to bind to head-to-head SOX10-binding sequences [21]. No other variants in the SOX10- and EGR2-binding sites listed in Table 1 were identified in our patient cohort.

The c.126-1086T>A variant is not present in the public SNP database (dbSNP), and we did not detect it in a panel of 514 chromosomes from a North America population. Analysis of additional family members revealed that c.126-1086T>A was inherited from the patient's father. However, the father was not available for clinical evaluation, therefore we are unable to conclude whether or not c.126-1086T>A segregates with disease. Examination of our multi-sequence alignment (Fig. 1C) and that available at the UCSC Human Genome Browser (Fig. 1D) revealed that the affected T nucleotide is extensively (although not fully) conserved among mammalian species. Of note, the two sources of armadillo sequence differed in the nucleotide at this position, being either an A (Fig. 1C) or a T (Fig. 1D); this discrepancy could represent a sequencing error, an alignment error, or a natural variation of this nucleotide in armadillo. These findings indicate that c.126-1086T>A is a rare variant within a non-coding genomic segment that may be important for *MPZ* function.

### c.126-1086T>A is associated with decreased *in vitro* enhancer activity

**Figure 2:**
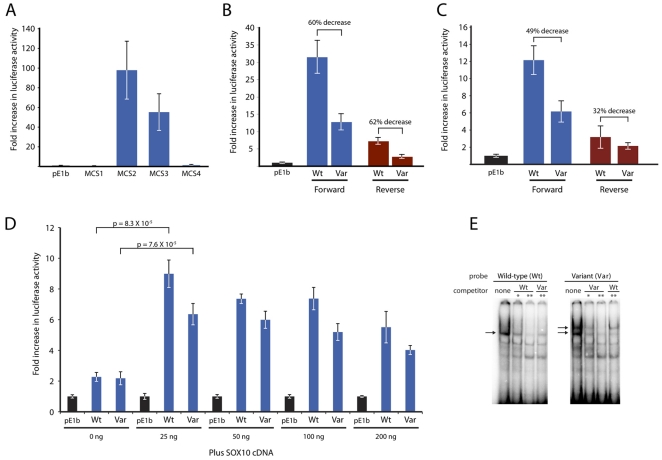
Functional characterization of the c.126-1086T>A variant in *MPZ*-MCS3. (**A**) Genomic segments spanning each *MPZ* MCS (*MPZ*-MCS1, *MPZ*-MCS2, *MPZ*-MCS3, and *MPZ*-MCS4) were tested for their ability to direct luciferase reporter-gene expression in cultured Schwann (S16) cells compared to a control vector (pE1b). Error bars indicate standard deviation. (**B**) Wild-type (Wt) and c.126-1086T>A variant (Var) forms of *MPZ*-MCS3 were tested for their ability to direct luciferase reporter-gene expression in the forward (blue) and reverse (red) orientations in cultured Schwann (S16) cells compared to a control vector (pE1b). Error bars indicate standard deviation. (**C**) Similar experiments as described in **B** were performed with cultured oligodendrocytes (OliNeu) cells. (**D**) Wild-type (Wt) and c.126-1086T>A (Var) forms of *MPZ*-MCS3 were tested for their responsiveness to SOX10 via luciferase reporter-gene expression in SOX10-negative MN1 cells, in the forward orientation compared to a control vector (pE1b). The amount of SOX10 expression vector co-transfected is shown. Error bars indicate standard deviation and p-values were calculated using the student's t-test. (**E**) Electrophoretic mobility shift analysis (EMSA) of wild-type and c.126-1086T>A alleles. ‘Probes’ (radiolabeled oligonucleotides) and ‘competitors’ (unlabeled oligonucleotides) corresponding to a 25-bp fragment including the wild-type (Wt) and c.126-1086T>A variant-containing (Var) consensus SOX10-binding sequence in *MPZ*-MCS3 (see Fig. 1B) were incubated with S16 nuclear extracts and subjected to electrophoresis (see Materials and Methods). Each assay used the same amount of probe coupled with varying amounts of competitor, as shown (‘none’ indicates no competitor; * and ** indicate a probe to competitor ratio of 1∶10 and 1∶100, respectively). Arrows denote specific nuclear proteins bound to the probe.

We tested each of the four conserved non-coding segments at *MPZ* (Fig. 1A) for their ability to direct reporter-gene expression (Fig. 2A). This involved cloning each segment upstream of a minimal promoter directing luciferase expression (Fig. 2A), transfecting each into SOX10-positive Schwann (S16) cells, and assessing luciferase activity as a proxy of reporter gene expression compared to a control vector with no insert (pE1b). MCS1 and MCS4 did not display any ability to direct reporter gene expression. In contrast, MCS2 and MCS3 directed reporter gene expression in a manner ∼90-fold and ∼45-fold higher than the control vector, respectively. Thus, these data confirm the previously-reported activity of the *MPZ* promoter (MCS2) and intronic enhancer (MCS3) *in vitro* and *in vivo* [11], [14], [15], [16], [17], further suggesting that these elements are required for *MPZ* expression.

To examine the effect of the c.126-1086T>A variant on *in vitro* enhancer activity of *MPZ*-MCS3, we cloned the wild-type and variant alleles (both forward and reverse orientations) upstream of a minimal promoter (pE1b) responsible for directing luciferase expression, transfected the resulting reporter constructs into cultured Schwann (S16) cells and oligodendrocytes (OliNeu) cells, and measured the luciferase activity relative to an internal renilla control. These studies revealed that the wild-type *MPZ*-MCS3 allele enhances luciferase expression by ∼31.5- and ∼7.2-fold (compared to a control reporter-gene construct lacking a cloned insert) in the forward and reverse orientations in S16 cells, respectively (Fig. 2B). In contrast, the c.126-1086T>A-containing *MPZ*-MCS3 allele enhances luciferase expression by ∼12.7- and ∼2.7-fold in the forward and reverse orientations in S16 cells, respectively (Fig. 2B). Thus, the c.126-1086T>A variant is associated with a 60–62% reduction of *in vitro* enhancer activity (depending on the orientation of the fragment in the reporter construct). Importantly, these data are consistent with previously-reported analyses on a similar construct with deleted SOX10 binding sites [16]. We also tested *MPZ*-MCS3 enhancer activity in OliNeu cells and found similar results as in S16 cells; the c.126-1086T>A variant is associated with a 32–42% reduction of *in vitro* enhancer activity, slightly less than observed in the S16 cells (Fig. 2C).

To determine the cell-type specificity of *MPZ*-MCS3, and to test the responsiveness of the wild-type and c.126-1086T>A alleles to SOX10, we tested the enhancer activity of each allele in SOX10-negative mouse motor neuron (MN-1) cells with, or without, co-transfection of a construct expressing SOX10 (Fig. 2D). First, neither wild-type nor c.126-1086T>A *MPZ*-MCS3 displayed significant enhancer potential in SOX10-negative cells. Second, the addition of 25 to 200 ng of a SOX10 expression construct dramatically increased enhancer potential (compared to the similarly-treated pE1b control vector), further suggesting that SOX10 specifically acts upon *MPZ*-MCS3. Interestingly, at each concentration of SOX10 expression construct, the mean value for the enhancer activity of the c.126-1086T>A allele (Var) was lower than that for the wild-type (Wt) allele (Fig. 2D); however, none of these experiments reached statistical significance.

### c.126-1086T> A is associated with altered nuclear-protein binding

SOX10 and EGR2 have previously been shown to bind to MCS3, and deletion of the SOX10 consensus sequences ablate SOX10 binding [16]. We used electrophoretic mobility shift assays (EMSAs) to establish if the c.126-1086T>A variant is associated with altered binding of nuclear proteins (e.g., transcription factors) to *MPZ*-MCS3. Briefly, ^32^P-labeled double-stranded oligonucleotides (*i.e.*, ‘probes’) containing the SOX10-binding consensus sequence within *MPZ*-MCS3 were incubated with nuclear extracts from S16 cells (pre-incubated with or without unlabeled oligonucleotides (*i.e*., ‘competitors’)), and then separated on a polyacrylamide gel. The probe containing the wild-type sequence appears to bind a single specific nuclear protein, while the probe containing the v c.126-1086T>A variant binds an additional nuclear protein (Fig. 2E). Notably, a competitor probe containing the variant sequence completely ablates binding of this additional nuclear protein, while an equivalent amount of the wild-type competitor did not. From this, we have shown that c.126-1086T>A alters the binding of nuclear proteins at *MPZ*-MCS3, potentially contributing to the reduced enhancer strength we observed in our *in vitro* studies.

### *MPZ*-MCS3 directs reporter-gene expression in developing zebrafish embryos

We further studied the function of *MPZ*-MCS3 *in vivo* using a zebrafish model system. We hypothesized that if *MPZ*-MCS3 contributes to *MPZ* transcriptional regulatory control, this sequence interval would likely direct reporter-gene expression in myelinating cells *in vivo*. Specifically, this would include central nervous system oligodendrocytes and peripheral nervous system Schwann cells. Tol2-harboring transgene constructs containing either wild-type or c.126-1086T>A human *MPZ*-MCS3 cloned upstream of a *c-fos* minimal promoter and the *EGFP* gene were injected into two-celled zebrafish embryos. We selected all embryos expressing *EGFP* in mosaic G_0_ embryos to be raised and screened for germ-line transmission of the transgene. *EGFP* expression was documented at selected developmental time points (ranging from 24 hpf to 7 dpf) in the offspring of multiple independent founder transgenic-zebrafish for each construct.

**Figure 3:**
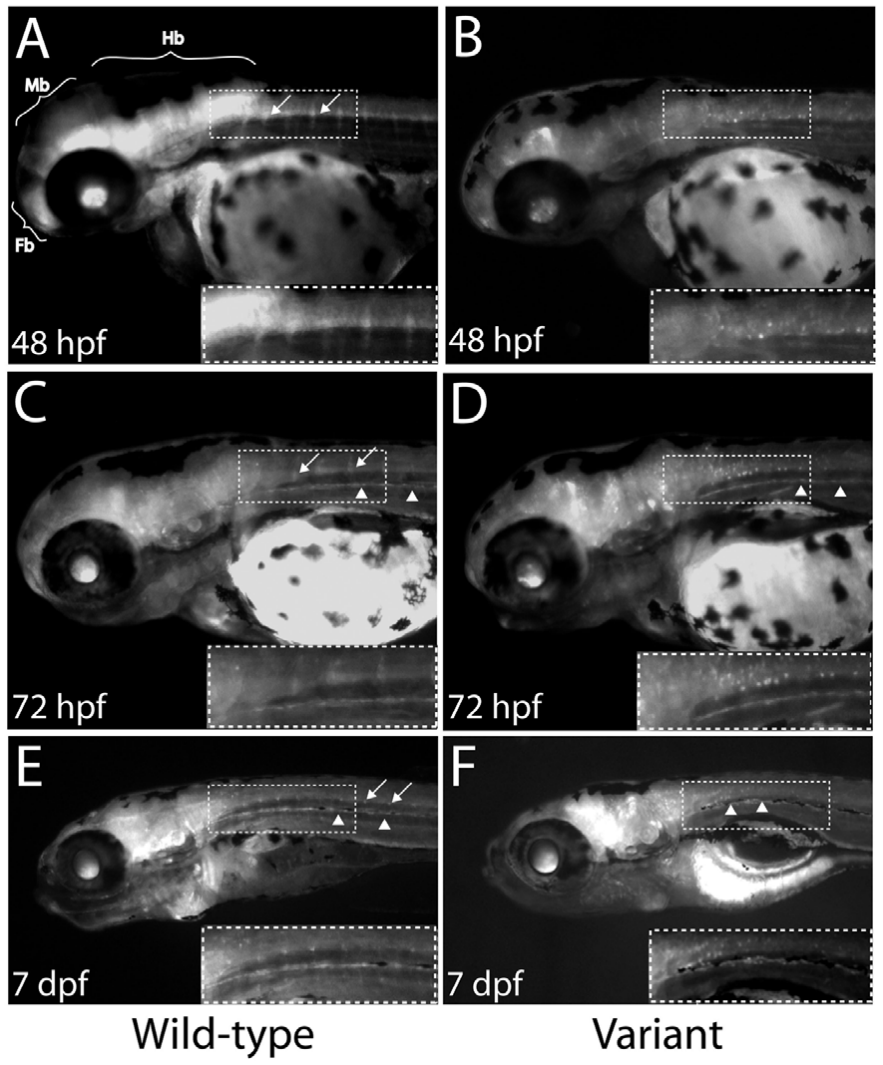
MPZ-MCS3 directs reporter gene expression in relevant tissues *in vivo*. Wild-type (**A**, **C**, and **E**) and c.126-1086T>A variant (**B**, **D**, and **F**) containing *MPZ*-MCS3 were studied for their ability to direct the expression of *EGFP* in developing zebrafish; expression was analyzed at the indicated developmental stages; *EGFP* expression is noted in structures consistent with the hindbrain (Hb), midbrain (Mb), forebrain (Fb), descending motor neurons (arrows), lateral line (arrow heads), and oligodendrocytes (boxes and inset in **F**).

Our analyses revealed strong EGFP expression in the telencephalon, midbrain tegmentum, lenses, and hindbrain (Fig. 3A, C, and E). At later stages (72 hpf and 7 dpf) expression became less abundant in the anterior CNS, yet remained present in the peripheral motor nerves; signal was also evident along the developing posterior lateral line nerve (arrow heads in Fig. 2C and E). This pattern largely correlates with endogenous *mpz* expression during zebrafish development—*mpz* is expressed in a dynamic spatial and temporal fashion from early stages in the lateral tegmentum, lens vesicles, optic nerves, hindbrain and anterior spinal cord [22], [23], [24]. Interestingly, *mpz* expression in zebrafish has been reported to be restricted to the CNS during early development [22], [25], whereas the mammalian orthologs are believed primarily to be expressed in Schwann cells [26].

In contrast to zebrafish harboring the wild-type enhancer, transgenic lines carrying c.126-1086T>A *MPZ*-MCS3 displayed markedly reduced EGFP expression in the forebrain, midbrain, and hindbrain (Fig. 3B, D and F), which was dramatically reduced at 48 hpf. Furthermore, descending motor nerves were not visible at this or other developmental time points in c.126-1086T>A *MPZ*-MCS3 zebrafish. While these differences may be due to the position of the inserted transgene and/or transgene copy number, they were observed in two wild-type and two c.126-1086T>A F1 zebrafish lines and are consistent with the above data showing decreased enhancer activity and altered nuclear-protein binding associated with the c.126-1086T>A variant (Fig. 2B-D). Both, mutant and wild-type enhancers displayed expression in the medulla oblongata and the spinal cord, potentially corresponding to the developing oligodendrocytic cells (Fig. 3B, DF and Fig. S1). In addition, we observed no overlap of reporter expression with acetylated tubulin (AcT) in the sympathetic nerves and probable oligodendrocytes in the spinal cord of 7 dpf fish carrying the wild-type *MPZ*-MCS3 (Fig. S1), which suggests that cells marked by this regulatory element are glial. Nevertheless, partial overlap of EGFP with AcT and mbp was visible in PLL ganglia (Fig. S1). This EGFP expression is consistent with glia ensheathing neuronal cells, nevertheless we cannot definitely exclude potential neuronal activity of the *MPZ*-MCS3 enhancer.

## Discussion

In this study, we show that a naturally-occurring, rare, non-coding nucleotide variant modulates the activity of an *MPZ*-transcriptional enhancer. Specifically, we performed targeted genomic re-sequencing of a collection of patients with neurological disease, focusing our attention on predicted SOX10- and EGR2-binding sites within evolutionarily conserved non-coding regions of the *MPZ* gene. These efforts resulted in the identification of a rare variant (c.126-1086T>A) in a previously described SOX10-binding site [11], [16] in the first intron of *MPZ*. We characterized this variant in greater detail using a number of methods, including human population screening, comparative sequence analysis, *in vitro* reporter-gene assays in cultured Schwann and oligodendrocyte cells, electromobility shift assays (EMSAs), and *in vivo* reporter-gene expression in zebrafish. Our data indicate that the c.126-1086T>A variant resides at a nucleotide position that is highly conserved among mammals, is associated with decreased enhancer activity and altered nuclear-protein binding, and is situated in a genomic segment that directs reporter-gene expression in relevant tissues in developing zebrafish.

We were unable to clinically evaluate the transmitting parent (father) of the patient carrying this variant (BAB1733). Therefore, the c.126-1086T>A variant was not implicated in disease onset in humans. Thus, the development of a relevant vertebrate model (*e.g.*, mouse) harboring c.126-1086T>A *MPZ* will be necessary to clarify the potential pathogenic role, if any, of this variant. However, our findings indicate that it would be prudent to screen these (as well as other) *MPZ* non-coding sequences for functional variants in human populations.

Our *in vitro* functional studies indicate that the wild-type genomic segment encompassing the c.126-1086T>A variant (*MPZ*-MCS3) directs reporter-gene expression in cultured Schwann and oligodendrocyte cells, does not display enhancer activity in SOX10-negative cells, is responsive to SOX10, and is bound by a nuclear protein—likely SOX10, which has been shown to bind this region in previous studies [11], [16]. Alternatively, c.126-1086T>A decreases enhancer activity in these same SOX10-positive cells and alters the binding of nuclear proteins. There are two possible interpretations of these data. First, it is conceivable the variant weakens the affinity for SOX10 and/or creates a novel protein binding site that competes with SOX10. Second, the variant could alter the nature of SOX10:EGR2 protein interactions in the nucleus; however, the fragment analyzed in EMSA analyses did not contain the EGR2 binding sequences. In any case, c.126-1086T>A *MPZ* represents a rare, functional variant this likely affects *MPZ* expression *in vivo*.

The studies we performed in zebrafish revealed that human *MPZ*-MCS3 directs reporter-gene expression in the hindbrain and spinal column in positions consistent with oligodendrocytes. These data are consistent with the established expression pattern of zebrafish *mpz* at 48 hpf and later [22]. Furthermore, human wild-type *MPZ*-MCS3 directs reporter-gene expression in distal regions of the descending spinal motor nerves, lateral line nerve, and sympathetic chain nerves. These findings are consistent with the expression of genes in Schwann cells, which myelinate all peripheral neurons. Interestingly, while *MPZ* is expressed in mammalian Schwann cells, *mpz* expression has not been detected in zebrafish Schwann cells up to 10 dpf by whole-mount *in situ* hybridization [22]. There are numerous possible explanations for this discrepancy. First, whole-mount *in situ* hybridization may not be sensitive enough to detect *mpz* expression in zebrafish Schwann cells. Second, *mpz* may be expressed at a later stage in zebrafish development, due to gene regulation by other elements (e.g., silencers) acting in a concerted fashion with the intron-1 enhancer. Third, *mpz* may not be expressed in zebrafish Schwann cells; in this case, the human wild-type *MPZ*-MCS3 transgene may direct expression of *EGFP* because *sox10* is expressed in Schwann cells in zebrafish by 30–40 hpf [27]. Finally, the discrepancy in expression patterns between fish and terrestrial vertebrates may be due to the early, rapid evolution of the myelination process, which is crucial for vertebrate development. In higher vertebrates, the major requirement for adhesive molecules in the CNS may be encoded by other loci [28]. Indeed, this might explain the EGFP expression observed in glial cells of both the CNS and PNS in our studies (Figs. 3 and S1)—while the transcriptional vocabulary may be preserved between human and zebrafish, the tissue-specific requirements and corresponding expression patterns may differ.

The first intron of *MPZ* shares little sequence conservation between mammals and zebrafish (Fig. 1A); indeed, it is not possible to generate a sequence alignment between mammalian species and zebrafish in this region (data not shown). This observation is surprising given that human *MPZ*-MCS3 is functional in developing zebrafish. Such a discrepancy has been encountered with other non-coding genomic segments [29], [30], and it has been proposed that current algorithms for detecting sequence conservation between evolutionarily diverse species are not sufficiently sensitive to identify all functional non-coding sequences [31]. There are two possible explanations for the surprising lack of sequence conservation in light of our finding that human *MPZ*-MCS3 directs reporter-gene expression in developing zebrafish. First, there may be functional conservation between the orthologous regions in mammals and zebrafish, with sequence conservation falling below a threshold required for computational detection; it is notable that there are a number of SOX10-binding consensus sequences within this genomic region in zebrafish (data not shown). Second, *MPZ*-MCS3 may be functional in mammals, but the orthologous region may not be functional in non-mammalian vertebrates (e.g., zebrafish); thus, *MPZ*-MCS3 might be a mammalian lineage-specific transcriptional regulatory element. Each of these possibilities could be investigated further by analyzing overlapping segments of the first intron of the zebrafish *mpz* gene for enhancer activity in appropriate cell culture and *in vivo* model systems.

The complete sequencing of the human genome [32] and the initial analyses that have subsequently been performed [33] reveal the complexities of non-coding DNA, including sequence elements that regulate transcription. The study of functional non-coding sequences is becoming increasingly relevant for understanding the genetic basis of human disease. Yet, major challenges remain both in identifying such sequences and in establishing if variants residing in such sequences are phenotypically relevant. While we were unable to implicate the MPZ intronic variant in disease onset, the experimental pursuits described here attempted to address the above-mentioned challenges. First, comparative sequencing coupled with other bioinformatic-based analyses were used to prioritize the more detailed examination of candidate functional non-coding sequences. Second, a variant detected in a human population was studied by both *in vitro* and *in vivo* assays in an attempt to experimentally establish the functional consequences of such variation. While *in vitro* reporter-gene assays are relatively routine, they do not provide broad biological insight into the role of the non-coding sequences being studied (or of the variants residing therein). Our use of zebrafish to study the wild-type and variant forms of *MPZ*-MCS3 nicely illustrates the value of an *in vivo* assay system, particularly since certain mammalian transcriptional regulatory elements are known to function in zebrafish. However, the zebrafish system also has limitations, such as for studying sequences that function in a mammalian- or human-specific fashion and for capturing quantitative data about reporter-gene expression. For the latter limitation, developing a zebrafish system for quantitatively measuring allelic differences in enhancer activity would be a major step toward improving our ability to characterize the functional consequences of non-coding variants and to establish their roles in human disease.

## Methods

### Ethics Statement

All of the studies performed herein were performed under IRB and ACUC approval from the National Institutes of Health (NHGRI), Baylor College of Medicine, and Johns Hopkins University.

### Comparative sequence analysis

Two complementary methods were used to identify multi-species conserved sequences (MCSs) in the genomic region encompassing the human *MPZ* gene. First, *MPZ*-containing bacterial artificial chromosomes derived from the following species were isolated and sequenced as described [34]: lemur (GenBank No. AC157839), dog (GenBank No. AC158443), cow (GenBank No. AC158441), hedgehog (GenBank No. AC157618), and armadillo (GenBank No. AC157449). Sequences encompassing *MPZ* in human [University of California at Santa Cruz (UCSC) Human Genome Browser March 2006 assembly, chr1:159521864-159550790], mouse (UCSC Mouse Genome Browser August 2005 assembly, chr1:170969846-171282175), rat (UCSC Rat Genome Browser June 2003 assembly, chr13:86986762-87476861), and zebrafish (UCSC Zebrafish Genome Browser July 2007 assembly, chr2:39940779-40174778) were obtained from the UCSC Genome Browser (genome.ucsc.edu). All sequences were analyzed using MultiPipMaker [pipmaker.bx.psu.edu/cgi-bin/multipipmaker [35] to yield a multi-sequence alignment (in the form of an acgt file), which was subsequently analyzed using ExactPlus [36] to identify segments at least 5 bp long and 100% conserved in at least 6 species. Second, the UCSC Human Genome Browser (March 2006 build) was used to identify non-coding regions in and around the *MPZ* gene (chr1:159521864-159550790) with a PhastCons score of at least 350. SOX10- and EGR2-binding sites were identified within the ExactPlus- and PhastCons-detected MCSs using the PATCH algorithm of the TRANSFAC database [37]; for this analysis, a minimum site length of 6 bp (tolerating zero mismatches with known binding sites) and a lower score boundary of 87.5 were required.

### Patient DNA samples and variant screening

DNA samples were obtained from patients diagnosed with neurological disease based on an examination by a collaborating physician. For genetic analyses, genomic DNA was purified from patient-derived peripheral blood or lymphoblastoid cell lines using standard procedures. All studies were performed with the approval of the appropriate Institutional Review Board.

Each genomic region of interest was screened for sequence variants by PCR amplification and direct sequencing of the amplified DNA segment. Specifically, oligonucleotide primers (sequences available upon request) were designed to amplify each region. PCR assays contained 1.5 µM of each primer, 0.2 µM dNTPs, 1.5 µM MgCl_2_, 1 unit AmpliTaq DNA Polymerase (Applied Biosystems, Foster City, CA), 1X of the manufacturer's buffer, and 200 ng of genomic DNA. Thermal cycling consisted of an initial denaturation for 2 minutes at 93°C, followed by 40 cycles of 93°C for 10 seconds, 55°C for 5 seconds, and 72°C for 30 seconds. A final incubation at 72°C was then performed for 7 minutes. Following electrophoretic separation on a 1% agarose gel, the PCR products were excised and purified using the QIAquick Gel Extraction Kit (Qiagen, Valencia, CA). DNA sequencing was performed using BigDye terminator chemistry. Reactions (20 µl total) consisting of 8 µl BigDye mix, 0.5 µl sequencing primer (at 25 µM), 6.5 µl water, and 5 µl PCR product (at ∼50 ng/µl) were subjected to 30 cycles of 92°C for 20 seconds, 55°C for 10 seconds, and 60°C for 4 minutes. Subsequently, unincorporated nucleotides were removed using CentriSep 8 column strips (Princeton Separations, Adelphia, NJ), and the final products were analyzed on a model 3100 DNA sequencing instrument (Applied Biosystems, Foster City, CA). Data were extracted and analyzed using Sequencing Analysis software (Applied Biosystems), and the resulting sequences were aligned with Sequencher (GeneCodes, Ann Arbor, MI). Genotyping for the c.126-1086T>A allele was performed by direct sequencing of PCR products (as above) amplified from DNA samples from a North American population [38].

### Reporter constructs

Reporter constructs for assaying enhancer activity and gene expression were generated using Gateway cloning technology (Invitrogen, Carlsbad, CA). Briefly, PCR primers containing flanking attB sites (on their 5′ ends) were designed to amplify each *MPZ*-MCS from human DNA. To obtain both the wild-type and c.126-1086T>A-containing alleles for the *MPZ*-MCS3 (see Results), PCR amplification was performed using a DNA sample from the heterozygous patient (BAB1733). PCR products containing each allele were then recombined into the pDONR221 vector (according to the manufacturer's instructions) to yield appropriate entry clones; the insert of each selected entry clone was sequenced to identify a clone containing each allele with no PCR-induced mutations. Subsequently, plasmid DNA purified from each entry clone was recombined with either the pLGF-E1b (for luciferase expression in cultured cells) [36] or pXIG-cfos-EGFP [for enhanced green fluorescent protein (EGFP) expression in developing zebrafish embryos [30]] destination vectors according to the manufacturer's instructions. Each resulting reporter construct was analyzed by restriction enzyme digestion (with BsrGI) to confirm the presence of an appropriately-sized insert.

### Assaying *in vitro* enhancer activity

Immortalized rat Schwann (S16) cells and mouse motor neurons (MN1) were cultured in Dulbecco's Modified Eagle Medium (DMEM; Invitrogen) supplemented with 10% fetal bovine serum (FBS; Invitrogen) and 1X glutamine, as previously reported [39]. Immortalized mouse oligodendrocyte (OliNeu) cells were cultured as previously reported [40]. Cells (5×10^4^) were placed into each well of a 96-well culture plate and transfected with luciferase reporter constructs (see above) using Lipofectamine 2000 reagent (Invitrogen, Carlsbad, CA). For each reaction, 0.25 µl Lipofectamine 2000 and 25 µl OptiMEM I minimal growth medium (Invitrogen) were combined and incubated at room temperature for 10 minutes. The purified luciferase reporter construct (200 ng) [or the equivalent volume of water (in the case of DNA-negative controls)] and 2 ng of purified pCMV-RL vector (a renilla-expression construct that served as an internal control; Promega, Madison, WI) were diluted in 25 µl OptiMEM I and then combined with the Lipofectamine-OptiMEM I mixture. The ∼50-µl reactions were incubated at room temperature for 20 minutes and then added to a single well of the 96-well culture plate containing cells. After a 3-hour incubation at 37°C, the medium was aspirated, and normal growth medium was added. The SOX10 expression construct was obtained from Origene (Rockville, MD).

Following a 48-hour incubation at 37°C, cells were washed with 1X PBS and lysed at room temperature using 1X Passive Lysis Buffer (Promega). An aliquot (4 µl) of the resulting cell lysate was transferred to a white polystyrene 96-well assay plate (Corning Inc., Corning, NY). Luciferase and renilla activities were measured using the Dual Luciferase Reporter 1000 Assay System (Promega) and a model Centro LB 960 luminometer (Berthold Technologies, Bad Wildbad, Germany). Each experiment was performed at least 16 times. In each case, the ratio of luciferase to renilla activity and the increase in this ratio over that measured for pLGF-E1b with no insert (indicated as ‘fold increase in luciferase activity’) were calculated. The mean (bar heights in Fig. 2) and standard deviation (error bars in Fig. 2) were determined using standard calculations.

### Assaying nuclear-protein binding

Complementary pairs of oligonucleotides (sequences available upon request) specific for the genomic DNA encompassing *MPZ* intron 1 SOX10-consensus sequence (see Results) were annealed and end-labeled with [γ-^32^P]ATP (PerkinElmer, Boston, MA) using 10 units of T4 polynucleotide kinase (Promega). The radiolabeled products (probes) were purified using an Illustra™ Microspin™ G-25 column (GE Healthcare, Piscataway, NJ). The ionizing radiation of each probe was measured using a liquid scintillation counter (L2 6500, Beckman Coulter, Fullerton, CA). Nuclear extracts from S16 cells, prepared using a Nuclear Extraction Kit (Cayman Chemical, Ann Arbor, MI), were incubated in a 20-µl reaction with or without unlabeled double-stranded oligonucleotides (competitor) in the presence of DNA-binding buffer (Promega) for 10 min at room temperature. Samples were then incubated with the above ^32^P-labeled probes (17.5 fmol/sample) for 20 min at room temperature. The DNA/protein complexes were separated on a 6% TBE DNA Retardation gel (Invitrogen) at room temperature, dried at 80°C for 1 hr, and visualized using a Fujifilm FLA-5000 image analyzer (FujiFilm, Tokyo, Japan).

### Gene-expression studies in zebrafish

Zebrafish were raised and bred using standard protocols, including maintaining staged embryos at 28°C [41], [42]. *EGFP*-expression constructs (see above) were injected into AB background G0 embryos (n≥200) as described [30], [43]. Injected embryos were evaluated for EGFP expression between 24 hours post fertilization (hpf) and 7 days post fertilization (dpf). Embryos showing consistent *EGFP* expression were selected and allowed to mature and subsequently crossed, which permitted germline transmission and better evaluation of *EGFP* expression. Embryos were imaged using a Carl Zeiss Lumar V12 Stereo microscope with AxioVision software (version 4.5).

7dpf embryos were anesthetized with tricaine (10 µg/ml) in embryo medium [42] and fixed in 4% paraformaldehyde in phosphate-buffered saline (PBS; pH 7.2) for 2 hours. They were then rinsed four times in PBST (PBS/0.1% Triton X-100), incubated in Proteinase K for 1 h at room temperature, washed 5×5 minutes in PBST, and incubated for 2 hours in blocking solution (10% goat serum, 1% bovine serum albumin [BSA], in PBST). Embryos were then incubated overnight at room temperature in primary antibody, rinsed 6×45 minutes in PBST 1% goat serum, and incubated overnight at room temperature in secondary antibody. They were then rinsed 5×10 minutes in PBST and transferred to 50% glycerol in PBS. For primary antibodies, anti-acetylated tubulin (Sigma) was used at 1∶100, anti-gfp (Invitrogen) was used at 1∶2,000. Alexa Fluor 488 and 555 (Invitrogen) were used as secondary antibodies (1∶1000).

## Supporting Information

Figure S1 MPZ-MCS3 wild-type transgenic zebrafish embryos (7dpf) stained for EGPF (A-K) and acetylated tubulin (AcT in B,C,E,F,H, and I) and MBP (K and L). Panels A-C and J-K display the lateral view at the PLL ganglion region. Panels D-F represent lateral views showing trunk expression, whereas panels G-I show sympathetic chain expression from the ventro-lateral view; anterior is to the left; white arrowheads indicate PLL; white arrows indicate probable oligodendrocytes and blue arrows point at cells expressing EGFP but not AcT in the sympathetic chain nerves.
(9.167 MB TIF)
