# Supplementary figures and images for: A Rare Myelin Protein Zero (MPZ) Variant Alters Enhancer Activity In Vitro and In Vivo
Source: PLoS One. 2010 Dec 16;5(12):e14346. doi: 10.1371/journal.pone.0014346 (PMC3002941; doi:10.1371/journal.pone.0014346)

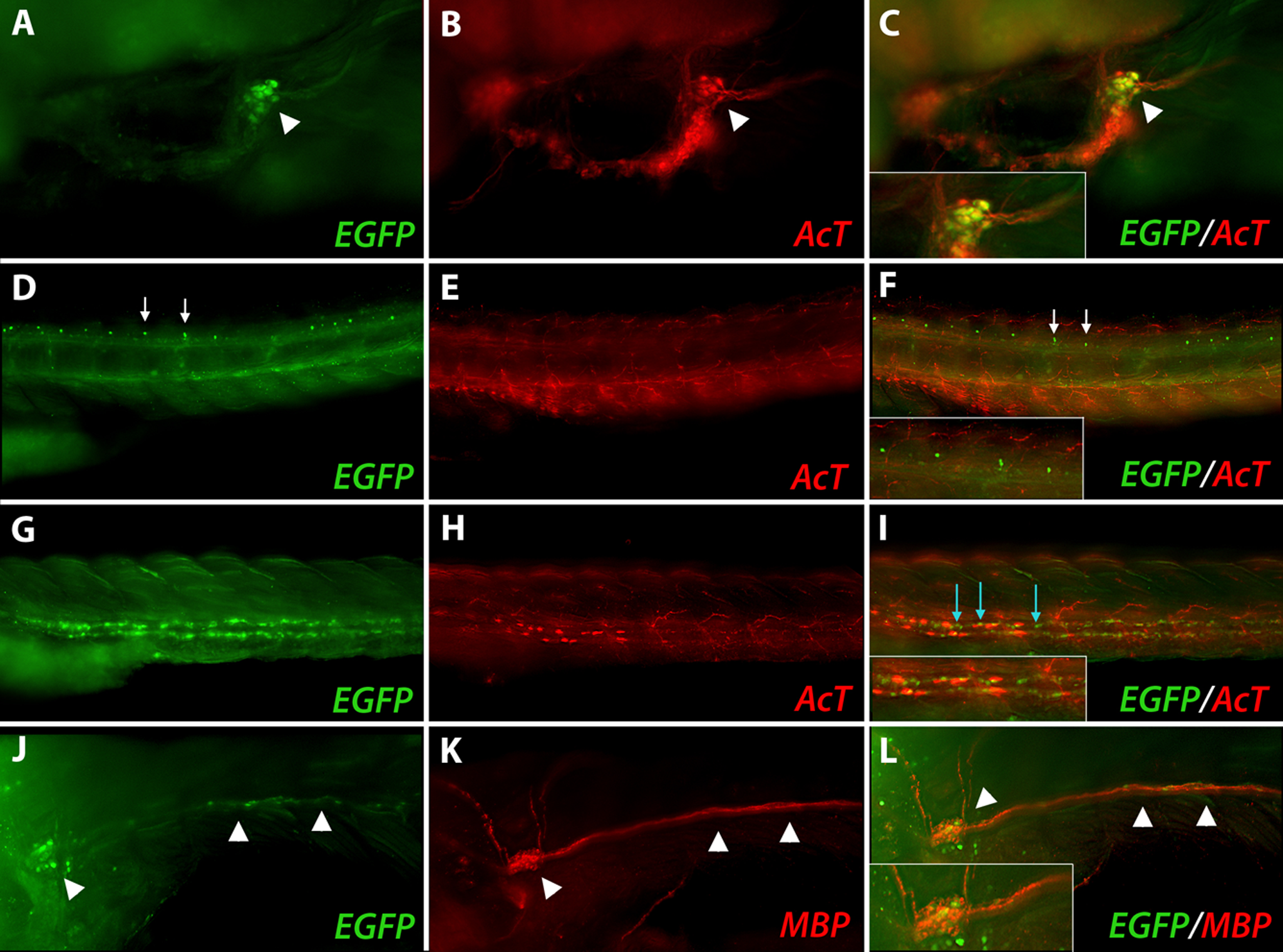

Supplement: Figure S1 — MPZ-MCS3 wild-type transgenic zebrafish embryos (7dpf) stained for EGPF (A-K) and acetylated tubulin (AcT in B,C,E,F,H, and I) and MBP (K and L). Panels A-C and J-K display the lateral view at the PLL ganglion region. Panels D-F represent lateral views showing trunk expression, whereas panels G-I show sympathetic chain expression from the ventro-lateral view; anterior is to the left; white arrowheads indicate PLL; white arrows indicate probable oligodendrocytes and blue arrows point at cells expressing EGFP but not AcT in the sympathetic chain nerves. (9.167 MB TIF) [file pone.0014346.s001.tif]
